# Supplementary material for: The interactome of intact mitochondria by cross-linking mass spectrometry provides evidence for coexisting respiratory supercomplexes
Source: Mol Cell Proteomics. 2017 Dec 8;17(2):216–32. doi: 10.1074/mcp.RA117.000470 (PMC5795388; doi:10.1074/mcp.RA117.000470)
Supplement: Supplemental Data [file supp_RA117.000470_133922_0_supp_23978_fzffwf.zip › spectra_annotation/mito_spectra_annotation/115-1-1-1-2-1.pdf]

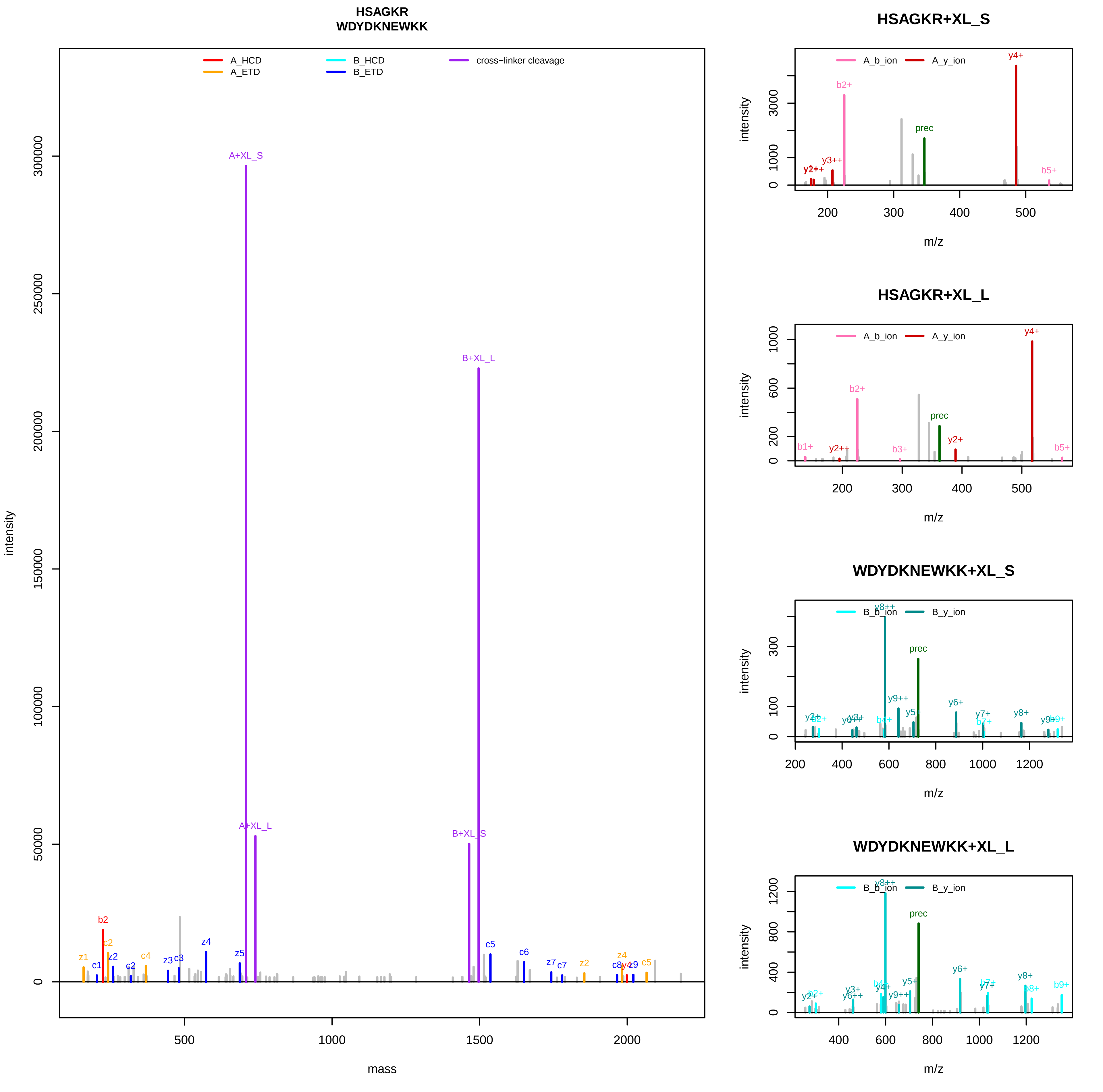
HSAGKR
WDYDKNEWKK
A_HCD
A_ETD
B_HCD
B_ETD
cross−linker cleavage
A+XL_S
B+XL_L
A+XL_L
B+XL_S
b2
c2
z1
c1
z2
c2
c4
z3
c3
z4
z5
c5
c6
z7
c7
z2
c8
y4
z9
z4
c5
intensity
mass
0
50000
100000
150000
200000
250000
300000
500
1000
1500
2000
HSAGKR+XL_S
A_b_ion
A_y_ion
y4+
b2+
prec
y3++
y2++
b5+
intensity
m/z
HSAGKR+XL_L
b1+
y2++
b2+
b3+
prec
y2+
y4+
b5+
WDYDKNEWKK+XL_S
B_b_ion
B_y_ion
y8++
prec
y9++
y5+
y6+
y7+
b7+
y8+
y9+
b9+
y2+
b2+
y6++
y3+
b4+
WDYDKNEWKK+XL_L
y8++
prec
y8+
b9+
b8+
b7+
y7+
y6+
y5+
y9++
y4+
b4
y3+
y6++
y2+
b2+
